# Minimally invasive surgery for pediatric renal and ureteric stones: A therapeutic update

**DOI:** 10.3389/fped.2022.902573

**Published:** 2022-08-18

**Authors:** Tao Peng, Hongcai Zhong, Baohui Hu, Shankun Zhao

**Affiliations:** ^1^Department of Pediatric Surgery, Huizhou Central People's Hospital, Huizhou, China; ^2^Department of Urology, Taizhou Central Hospital (Taizhou University Hospital), Taizhou, China

**Keywords:** urolithiasis, pediatric, update, treatment, surgery

## Abstract

The incidence of pediatric urolithiasis (PU) is growing worldwide. The corresponding therapeutic methods have become a research hot spot in pediatric urology. PU has the characteristics of abnormal metabolism, easy recurrence, and immature urinary system development, which make its treatment different from that of adults. Pediatric urologists should select the optimal treatment modality to completely remove the stones to prevent recurrence. Currently, the curative treatments of PU include extracorporeal shock wave lithotripsy (ESWL), ureteroscopy, retrograde intrarenal surgery, percutaneous nephrolithotomy (PCNL), laparoscopic, robot-assisted laparoscopic, and open surgery. This review aims to conduct a therapeutic update on the surgical interventions of both pediatric renal and ureteric stones. It accentuates that pediatric surgeons or urologists should bear in mind the pros and cons of various minimally invasive surgical treatments under different conditions. In the future, the treatment of PU will be more refined due to the advancement of technology and the development of surgical instruments. However, a comprehensive understanding of the affected factors should be taken into account by pediatric urologists to select the most beneficial treatment plan for individual children to achieve precise treatment.

## Introduction

With the changes in the dietary structure and lifestyle, as well as with the help of early detections, the incidence of pediatric urolithiasis (PU) is rapidly growing worldwide (1). As a result, therapeutic methods have become a research hot spot in pediatric urology. Due to the anatomical and physiological characteristics of the urinary system in children, the etiology and treatment of PU are different from those in adults. The main causes of PU commonly include congenital malformations of urinary tract anatomy, metabolic abnormalities, and urinary tract infections. Obstruction of ureteropelvic junction (UPJO), giant ureter, ureteral cyst, and urethral valve greatly contribute to the anatomic abnormalities of PU. Metabolic abnormalities predominantly include hypercalcinuria, hypocitric aciduria, hyperoxaluria, hyperuricuria, hypouricuria, cystinuria, and hypomagnesuria (2, 3). Preventive therapies (i.e., potassium citrate) and non-specific prophylactic treatments (i.e., refrain from animal proteins, salt, and simple sugars; and increase water intake) could prevent the reformation of stones in children (4).

Pediatric patients are prone to urolithiasis recurrence, thus every effort should always be made to reduce or prevent stone recurrence. In addition, reducing the number of treatments and minimizing the adverse effects on the developing kidneys are also important. In the past decades, the treatment methods for PU have changed dramatically because of the development of equipment, technological innovation, and the increasing experience of pediatric urologists. The original traditional open surgery is replaced by the current minimally invasive surgeries, including extracorporeal shockwave lithotripsy (ESWL), ureteroscopy (URS), and retrograde intrarenal surgery (RIRS), percutaneous nephrolithotomy (PCNL), laparoscopic, and robot-assisted surgery (5, 6). Since there are many minimally invasive treatment methods, pediatric urologists must be familiar with the advantages and shortcomings of each treatment option. In this comprehensive review, we endeavor to summarize the current status and recent advances in surgical management options in PU.

## Literature search

To maximally find the relevant studies that met the topic of surgical management options in treating PU, we performed a comprehensive review on the most common-used databases, e.g., MEDLINE, EMBASE, Google Scholar, and Cochrane Library. The keywords searching strategy in the MEDLINE included ESWL, PCNL, standard-PCNL, Mini-PCNL, Ultra-Mini-PCNL (UMP), super-mini-PCNL (SMP), Micro-PCNL, ECIRS, URS, RIRS, laparoscopic, and robotic treatments in PU.

### ESWL

In 1986, Newman et al. presented the first report on ESWL in treating PU (7). Until now, ESWL is still the first-choice treatment for managing most renal and ureteral stones in children (8). It is suggested that ESWL is the first-line treatment option for pediatric renal stones <1.0 cm in diameter, regardless of the Hounsfield unit (HU) value. In addition, non-lower calyx renal stones with a HU value of <750 and a diameter ranging from 1.0 to 2.0 cm are also recommended. To treat the stones in the upper ureter with a diameter of <1.5 and <1.0 cm in the middle or lower ureter, ESWL can be given priority (9–11).

In terms of urolithiasis, ESWL has numerous strengths in children than adults. First, the skin-to-stone distance in children is shorter than in adults, and the shock wave energy attenuation is reduced. Second, the water content of the tissue between the children's body surface and the kidney tissue is higher, and the acoustic impedance is low, which is also conducive to energy transmission. Third, the children's ureter is shorter and the gravel is discharged easier (12).

Due to the different types of lithotripters, the number, frequency, and energy used during treatment, the stone clearance rate (SFR) reported in the different studies is inconsistent. Currently, several types of shock wave generation are available, including electrohydraulic, electromagnetic, and piezoelectric (13). It was reported that there was no significant difference in treatment outcomes between electromagnetic and electrohydraulic methods (14). According to the results from both clinical and experimental researches, the commended shockwave frequency for PU is 60/min (1 Hz) (15). The SFR was about 90% for the renal pelvis and upper ureteral stones, while 50–62% for the lower calyx stones (16).

The main defect of ESWL is necessary for several treatment sessions. Besides, a proportion of the patients need additional auxiliary procedures to achieve complete stone clearance. The main contraindications for ESWL in children are coagulation dysfunction, cardiopulmonary disease, lithotripsy passage obstruction, uncontrolled urinary tract infection, diabetes, skeletal malformations, excessive obesity, active infectious diseases, renal insufficiency, and hemangioma around the stone (6, 17).

The children's age, body mass index, stone size and location, hardness of stone, anesthesia effect, operator's skills and experience, and anatomical structure of the renal calyx are the main factors that affect the result of ESWL. Alsagheer et al. (18) reported that ESWL was more successful in younger children. The author further pointed out that age was the only independent predictor of surgery success when conducting a multivariate analysis. ESWL success rates are lower in adult obese patients, but obesity has not been shown to significantly affect the success of fragmentation in ESWL in children (19). Kizilay et al. (20) found that obesity is an important factor in ESWL success in multivariate analysis. The EAU guidelines 2022 (21) showed that renal pelvis and upper calyx stones respond well to ESWL. The SFR was up to 90% for both the renal pelvis and upper ureteral stones (12). Irrespective of the location, as the stone size increased, the SFR decreased and the re-treatment rate increased. As reported, the SFR in children receiving ESWL with stone diameters <1, 1–2, and >2 cm were 90, 80, and 60%, respectively. Besides, with the increase in stone size, the rate of ESWL retreatment and radiation exposure may also increase, which may result in adverse effects on children's growth and development. The responses of cystine, calcium phosphate, and calcium oxalate monohydrate stones to ESWL are poor (22). Pediatric patients known to have these stone compositions might be better directed to other treatment alternatives. El-Assmy et al. (23) concluded that HU value ≤ 600 HU and stone length ≤ 1.2 cm were significant independent predictors of ESWL success when treating PU. ESWL treatment in children is commonly conducted under general anesthesia. Under anesthesia, the high-frequency shallow ventilation might reduce the range of respiratory movement, keeping a relative fixed position of the stones. Increasing the hit rate of the shockwave is recommended to reduce the apprehension, pain, and movement of a child. Besides, it can also consistently keep the stone under the shockwave target (24).

With the improvement of modern (second and third generation) lithotripters, a growing number of children use ESWL to treat upper urinary stones. To check whether the transmission zone is free of air bubbles and if the coupling is optimal, the ESWL devices are gradually accompanied by a video camera (15). This equipment can be incorporated into the therapy head of some lithotripters. Furthermore, other strengths are the identification of the target and maintenance of an optimal position of the patient throughout the procedure by using real-time ultrasound or continuous fluoroscopy (25). Of note, due to the poor radiopaque appearance on fluoroscopic imaging, ultrasound-guided had a higher success rate than fluoroscopic-guided ESWL when treating pediatric cysteine stones (26).

After ESWL, several complications, such as hematuria, renal hematoma, renal ureteral injury, renal colic, stone street formation, and urinary tract infection, may occur (27). The most frequent complication after ESWL treatment is ureteric obstruction induced by the stone fragments. Large stone burden and the fragments significantly increase this complication (28). Another common complication is urinary tract infection, which occurs secondary to the infected stone or urine. However, preoperative antibiotics based on the culture and sensitivity reports may be effectively avoided urinary tract infection. It should be admitted that special attention should be paid to the occurrences of renal subcapsular hematoma, renal parenchymal impairments, and surrounding tissues' injuries. Subcapsular hematoma is mainly induced by the application of an excessive number of shockwaves. In addition, unnecessarily enhancing energy levels for breaking the stone in a single session may also attribute to subcapsular hematoma. It was suggested that ramping energy modalities might improve stone fragmentation, resulting lower incidence of hematoma and kidney injury (29). Therefore, clinicians should conduct lithotripsy with a safer method by reducing the excessive numbers of shockwaves as well as the high-risk energy levels (15).

ESWL also has many issues that are worthy to further research and discussion. First, the ESWL technology is well established, thus it is difficult to further improve the ESWL outcome of renal stone management. On the other hand, ESWL failed to keep pace and compete with the better outcomes of the newer minimally invasive surgeries. Second, whether the Double J (D-J) tube needs to be indwelled in advance before the start of ESWL still needs discussion. Torricelli et al. (30) suggested that there is no need for routine indwelling D-J tube before ESWL treatment. D-J tube needs to be indwelled before treatment only when the child has renal insufficiency or isolated kidneys. Third, it is necessary to further clarify whether ESWL will cause long-term adverse effects on the developing kidneys. In 2013, EI-Nahas et al. (31) conducted a long-term follow-up of children after ESWL and found that kidney development was normal, and there were no secondary hypertension and diabetes. In the future, large-scale prospective RCTs with long-term follow-up are still warranted to further determine the exact impact of ESWL on children's developing kidneys. Fourth, perioperative antibiotic prophylaxis for ESWL, either adult or pediatric patients, was still controversial. A multicenter study showed that the usage of prophylactic antibiotics was not independently correlated with post-ESWL urinary tract infections (OR = 1.269, 95% CI: 0.886–1.818, *P* = 0.194) (32). Therefore, routine antibiotic prophylaxis was not required for patients undergoing ESWL (33). However, a single short of antibiotics is recommended in those with positive urine culture and it is a need to treat with antibiotics at least 24 or 48 h before the procedure.

### PCNL

In 1985, PCNL was first used in pediatrics (34). Classical PCNL, also named standard PCNL, in children, required a 30Fr Amplatz sheath and applied a 24-Fr nephroscope. Since then, many studies have reported that using adult-sized surgical equipment to perform PCNL for children, the postoperative SFR is satisfactory and the complications are within an acceptable range. The EAU guidelines 2021 recommend that PCNL can be used to treat children with complete or partial staghorn stones, kidney stones >2.0cm in diameter, lower calyx stones >1.0cm in diameter, and kidney and ureteral stones, which have failed ESWL and RIRS. And the EAU guidelines 2021 also emphasized that contraindications include uncorrected systemic bleeding disorders, untreated/uncontrolled urinary tract infections, tumors in the presumptive access tract area, and suspect malignant renal cancers (21, 35).

With the advancement of technology, the development of equipment, and the accumulation of experience of pediatric urologists, multiple classifications have been suggested and published in the literature, which include standard PCNL (24-30Fr), mini-PCNL (14-20Fr), ultra-mini-PCNL (11-13Fr), and super-mini-PCNL (10-14Fr) (Table 1). Due to miniature equipment for PCNL being performed in pediatric patients, these PCNL techniques could not only be applied in all age groups but also present an opportunity to deal with smaller stones that would otherwise be candidates for ESWL or RIRS.

**Table 1:** Current evidence of miniaturized-PCNL and RIRS procedures in pediatric patients.

**PCNL technique**	**Access sheath (Fr)**	**Mean stone size (mm)**	**Stone site**	**Stone-free rate**	**Time of the procedure**	**Mean drop in Hb** **(g/dL)**
Standard-PCNL	24–30	>20	Renal stones	56–100	74.7–118.9 min	0.97–3.5
Mini-PCNL	14–20	<30	Renal stones	76–100	58–122 min	0.23–8.9
Ultra-mini-PCNL	11–13	<25	Renal stones	88.9–97.5	24.5–93.5 min	0.2–0.9
Super-mini-PCNL	10–14	<25	Renal stones	94.8–98.7	25–36.4 min	0.3–1.0
Micro PCNL (Microperc)	4.85	<20	Renal stones	80–100	37.2–83 min	0.5–3.0
RIRS	Ureteral access sheath: 9.5–12	<20	Renal and ureteral stones	84.3–97	47.5–109.7 min	Total complication rate: 2–8%

PCNL, percutaneous nephrolithotomy; RIRS, retrograde intrarenal surgery. The above data were derived from the following publications (36–50).

### Standard PCNL

The traditional standard PCNL channel size is 24Fr to 30Fr. The earliest research reported that standard PCNL treatment for pediatrics' urolithiasis, the use of adult-sized equipment for children, the postoperative SFR is about 47–90%, and the complication rate is low (51). The main strength of standard PCNL with such large access is high stone clearance rates (>90%) in a single session. However, it is difficult to apply this technique to children due to the potential risk of renal damage or excessive bleeding. Bilen et al. (52) compared the results of PCNL in children with urolithiasis using three sizes of working sheaths. The results showed that the SFR of children using the 26Fr working sheath was 69.5%, the 20Fr working sheath group was 80%, and the 14Fr working sheath group was 90%. However, the rate of blood transfusion treatment in the 26Fr and 20Fr working sheath groups has increased, and there are no children in the 14Fr working sheath group who needed blood transfusion (52).

### Mini-PCNL

To further improve the safety of PCNL, urologists began to perform minimally invasive PCNL. The miniaturization of equipment for PCNL also facilitated its use in all age groups. In 1997, Helal et al. (53) first reported that they used a 15Fr working sheath and a 10Fr children's cystoscope to treat pediatrics' urolithiasis. Mini-PCNL is a modified standard PCNL procedure that uses a smaller channel (14Fr to 20Fr). The instruments involved are composed of an 8.0/9.8Fr semi-rigid ureteroscope or an 8.5/12.5Fr mini nephroscope, and a pulsatile high-pressurized endoscopic perfusion pump. The scaled fascial dilators are used for dilating the percutaneous tract, starting from 8Fr and to be scaled up to 14-20Fr (54). Under this technique, most of the stone fragments could be pushed out *via* the sheath with a pulsed perfusion pump. This pump can pressurize up to 300 mmHg for about 3 s, and then pause for 2 s, before the cycle repeats. A fast removal of the endoscope leaving the sheath contemporized with the low-flow irrigation period generates a relative vacuum within the sheath. These, together with the coil of the system from the transient high pressure caused by the irrigant, stone fragments can be effectively flushed out. This technique could significantly shorten the operative time due to the stone fragmentation can be removed continuously with little downtime for the instrument. Moreover, the pressurized irrigation hardly induces dangerously high intrapelvic pressure (55). Currently, different types of energy for intracorporeal lithotripsy during PCNL are available, including ultrasonic and pneumatic systems, trilogy, and holmium or thulium laser.

Zeng et al. (56) first performed Mini-PCNL in 20 children, the final SFR was 95%, and there were no children who need a blood transfusion during the perioperative period. At the same time, they evaluated the renal function preoperative and postoperative of the PCNL procedure and found stabilization of the glomerular filtration rate in the affected kidney. ElSheemy et al. (57) also suggested that superior outcomes with mini PCNL (14Fr) for renal calculi of 10–25 mm in preschool children compared with ESWL, while the complication rates were comparable.

The mini-PCNL's advantage is that it can further reduce the incidence of bleeding and other complications, relieve perioperative pain, shorten the length of hospital stay, and reduce the cost of treatment. Rashid et al. (58) have analyzed prospective data of 28 children undergoing mini-PCNL. They found that the initial SFR was 78%, which elevated to about 90% following a few ancillary procedures. And the SFR was detected inversely based on the stone burden and stone complexity (all *P* < 0.05). The complications were significantly associated with the stone complexity and the number of tracts (all *P* < 0.05). Onal et al. (59) demonstrated that the application of a sheath size >20Fr was an independent factor for predicting the complications and the bleeding necessitating transfusion in pediatric patients who underwent PCNL. Also, a larger tract was reported to be correlated to greater blood loss (48). Thus, patients with mini-PCNL may have shorter durations of hospitalization due to this technique may remarkably reduce patient discomfort and achieve lower peri-operative morbidity by using a smaller and less traumatic nephrostomy tract.

### Ultra-mini-PCNL

To further improve the safety and effectiveness of Mini-PCNL and reduce the incidence of complications, Desai et al. proposed a new minimally invasive PCNL procedure and named it Ultra-Mini-PCNL (UMP) (60). UMP makes use of a 3Fr telescope with a 7.5Fr nephroscope and an 11-13Fr sheath. This smaller tract size results in a reduction in cross-sectional surface area to about 1/8 of the original tract size used in conventional PCNL (30Fr). Such a small tract size may decrease the risks of bleeding and tissue trauma. Desai et al. (61) found that UMP could achieve one-step expansion when expanding the fascia, shortening the intraoperative radiation exposure time. The cross-section of the puncture tract is only about 30% of the standard PCNL. The working sheath of the UMP contains a tube with 3Fr welded to the inner wall before being linked to a port externally, which allows stone fragment retrieval without applying a grasper. The indications for UMP commonly included four aspects: 1) moderatesized stones as an alternative to ESWL or RIRS, 2) low pole stoneswhich were not amenable to RIRS, 3) diverticular renal stones, 4) stones refractory ESWL (60). With advances in laser technology, it has been readily utilized in various types of PCNL techniques (62). In general, for UMP in a pediatric population, stones could be fragmented by using Holmium: YAG laser (power up to 60 W) (40).

UMP is confirmed to be correlated with a high SFR, a low complication rate, and a low incidence of additional auxiliary procedures. It has advantages in treating stones <20 mm in diameter located in the lower pole calyx (63). As compared with ESWL, UMP has strength in the treatment of lower calyx stones that fragments hard to pass, such as in the long and narrow calyces and a sharp angle. Wilhelm et al. (64) compared the outcomes of UMP and RIRS for treating renal stones with 10–35 mm and observed that both techniques achieved high SFRs and low complication rates. As reported, the incidence of complications (e.g., hematuria, renal extravasation, or renal pelvic perforation) caused by UMP was recorded at 14%. Most of them were Clavien grade I and II complications, while there were no grade IV and V complications (65). This is of even higher importance in pediatric populations where a decrease in hemoglobin carries a greater physiological effect and could be fatal.

Because there is less bleeding during the operation, it also makes the nephrostomy tube unnecessary. In addition, when the ultra-mini-access is inadequate for the surgery procedure, conversion to 14-20Fr tract of mini-PCNL by enlarging the dilation is easy, due to the channel access has already been set up. However, due to the narrowing of the channel, UMP does not allow the use of forceps to retrieve the gravel during UMP, which would prolong the operation time (60). Dede et al. (66) reported the results of UMP treatment for 39 children with kidney stones. The results showed that the final SFR was 87.1%, and there are no children who received a blood transfusion during the perioperative period. They also concluded that UMP not only guarantees a high SFR but also reduces intra-renal pressure during surgery, which improves the safety of surgery.

### SMP

In recent years, the technological development of miniaturized PCNL procedures has been remarkable. These outstanding techniques with small percutaneous tract sizes can reduce the bleeding and continue to maintain a high SFR. However, some drawbacks of these techniques should be acknowledged, e.g., lower irrigation flow, poorer endoscopic visualization, less stone fragment extraction, and the potential risk of high renal pelvic pressure during irrigation. To further optimize the PCNL technique, Zeng et al. (67) designed a novel miniature endoscopic system to improve the safety and efficacy of the present PCNL technique and named this technique SMP. SMP system consists of an 8.0Fr nephroscope and a novel irrigation-suction sheath. The working diameter of the telescope of SMP is only 1.4 mm, but is characterized by a 40,000-pixel resolution. The sheath of SMP was designed with a two-layered metal structure, which was available in either 12 or 14Fr. There are two layers in the sheath, containing an independent irrigation channel and a conduit for suction (67). As reported by Zhao et al. (68), SMP is suitable for pediatric patients with stones size <2.5 cm with previously failed ESWL. The new design of SMP can remarkably prevent excessive intrarenal pressure, improve the visualization, as well as accelerate the stone fragment extraction. The new-generation SMP enables surgeons to introduce larger working instruments, e.g., 550 μm laser fiber and 1.0 mm lithotripter (69).

The main difference points between first-generation SMP and the UMP is the management of stone fragments. In UMP, similar to other previous PCNL technique, the stone fragments can be flushed out by the recoil of pressurized irrigation together with the action of the removal of the endoscope out of the working sheath. In SMP, the stone fragments can be removed by negative pressure aspiration. Simayi et al. (70) demonstrated that SMP could remove larger stones than mini-PCNL for treating PU (2.0 vs. 1.5 cm, *P* = 0.001). In addition, the authors also found that SMP was significantly correlated to a shorter postoperative hospital stay, and a higher tubeless rate (all *P* < 0.05).

There are several strengths when using the SMP technique. First, both stone fragmentation and dusting can be extracted effectively. Second, the visual field is clear during continuous irrigation. This is because the novel irrigation system could minimize the “dust storm” and bleeding. Third, a low average renal pelvic pressure was maintained throughout the procedure on account of the negative pressure aspiration facilitating irrigation drainage. As a result, SMP could significantly prevent sepsis, which was frequently caused by excessive renal pelvic pressure intraoperatively (71). Fourth, the SMP is designed by using medically-graded steel instead of plastic, it could decrease renal parenchymal trauma. This is because the traditional flexibility induced by a plastic sheath might allow the nephroscope to bent excessively, thus leading to scope damages (67). As expected, SMP may fill the gap between RIRS and conventional PCNL or replace RIRS.

Liu et al. (72) reported that SMP was performed on 111 children. The stones were all located in the lower calyx of the kidney with an average size of 1.4 cm. The final SFR was 90.1%, and the complication rate was 15.3%. Both of them were Clavien grade I and II complications. The most common complication was low fever. All recovered after symptomatic treatment (72). In some respects, SMP may potentially benefit for pediatric patients when compared to RIRS. It was suggested that RIRS might cause a small but not insignificant risk of injury to the delicate pediatric ureters (73).

In those selective pediatric patients who underwent SMP treatment, a high SFR can be achieved *via* a single treatment session. In addition, SMP is also associated with a high totally tubeless rate and less auxiliary procedures to clear stones. Moreover, the complication rates are comparable with other techniques when managing stones sizing <25 mm (74). Though SMP can be applied in managing larger stones, the selection of SMP should be prudent, which may be beneficial to achieve a successful treatment for PU.

### Micro-PCNL (microPERC)

Desai et al. (75) described a novel variation of the mini-PCNL that he called the Microperc, wherein renal access is accomplished in a single step with the use of an all-seeing needle with a 4.85F tract size. This miniaturized-PCNL method has applications not for only adult patients but also for pediatric patients. The main application of micro-PCNL in children is to minimize bleeding. In addition, with no need for tract dilatation, less radiation exposure, and, consequently, less operating time, it results in lower complication rates. The specific indications for patients treated with micro-PCNL are those considered unsuitable for ESWL secondary to stone composition or unfavorable pelvicalyceal system anatomy (50). However, some limitations should be acknowledged, including the vision was not as clear as other mini PCNLs, difficult to access a different calyx when stone fragments were migrated, and the expensive microperc instruments (5). Micro-PCNL does also not allow for the removal of bigger stone fragments and these are therefore left to pass spontaneously (65). Besides having no stones for biochemistry, this can also lead to re-admission with renal colic secondary to ureteric obstruction. Collection of urine post-operatively to sieve fragments is possible but is not always achievable and many patients may not comply.

### The potential point of concerns

The adverse effects of PCNL on children mainly include damage to the kidney parenchyma and function, intraoperative radiation exposure, and surgical-related complications. Dawaba et al. (76) followed up 65 children with urolithiasis treated with PCNL for a long period. No renal scarring was observed in these patients by detecting with a dimercaptosuccinate (DMSA) renal scan. At the same time, GFR has improved in all children after surgery (76). Miniature instruments are costly and meant to be disposable, making their universal use in developing countries, where much of the stone burden is on children, extremely difficult. And urologists should pay attention to the effects of perfusion fluid and operating room temperature on the body temperature of children during the operation to avoid excessive energy loss. During the operation, it should be noted that children are low in weight and have poor tolerance to blood loss. In conclusion, we recommend that pediatric urologists are proficient in performing PCNL for adults and have accumulated sufficient experience before starting PCNL for children.

### URS and RIRS

Marshall (77) first introduced the concept of endoscopic access to the renal collecting systems for managing upper urinary tract diseases with a rudimental fiberscope. Later, Richie et al. (78) tried to apply URS to extract lower ureteric stones in children in 1988. Currently, the URS equipment continues to be adapted for treating PU.

URS is particularly suitable for managing urinary calculus in the distal and mid ureter and is superior to ESWL (79). Semi-rigid URS with the size of 4.5/6Fr, 6/7.5Fr, and 8/9.8Fr can be applied depending on the age and anatomy of the children. Other influence factors include the stone size, location of the stone, and technical requirements. As compared to the fully flexible models, the semirigid URS are more durable and visible, combined with faster irrigation flow and larger working channels. Thus, semirigid URS can access the whole ureter and even the pelvicalyceal system. However, the capability of this scope to bend is limited. So, it is difficult to access the upper ureter as compared with flexible URS, especially in those patients with large psoas muscles (80). Benefiting from the deflectable tip of the flexible scopes, flexible URS is more suitable for tortuous ureter and upper ureteric stones.

RIRS is recommended to treat upper ureteric and renal stones ≤ 2cm (81), while FLURS allows better treatment and access to lower pole urolithiasis due to its greater flexibility, maneuverability, and secondary deflection capability, and wide range of deflection (82). Li et al. (83) performed the RIRS in 55 infants with upper ureteric and renal stones and found that the SFR was up to 94.6% without serious complications associated with this technique. Resorlu et al. (84) have studied and compared the effects of Mini-PCNL and RIRS for children with kidney stones. They performed Mini-PCNL on 106 children; and 95 children performed RIRS. The results showed that the SFR of children who underwent Mini-PCNL was 86%, while the SFR of children who underwent RIRS was 84%. However, the complication rate of children in the RIRS group was significantly lower than that of the Mini-PCNL group. All complications were Clavien grades I and II, and all of them recovered after conservative treatment. At the same time, they believe that compared to Mini-PCNL, RIRS reduces the risk of intraoperative radiation exposure and shortens the operation time. A randomized trial developed by Saad et al. demonstrated that mini PCNL had better SFRs than RIRS (95 vs. 71%, *P* = 0.046) in pediatric patients with stones >2 cm (85). However, RIRS was associated with less radiation exposure and fewer complications. Besides, RIRS is performed in the natural route of the urinary tract, minimizing the trauma to the tissues.

Although RIRS has been proven by a large amount of literature to be a safe and effective operation in the treatment of urolithiasis for children, it also has contraindications. It was suggested that the contraindications of RIRS for children include that uncorrected systemic bleeding disorders, severe cardiopulmonary insufficiency and intolerance of surgery, uncontrolled urinary tract infection, severe urethral stricture, and gross hematuria, narrow ureter, lower calyx stones, and IPA <30° (21). And there are certain limitations with RIRS in children. Due to the narrow caliber ureters in the children, access is difficult for RIRS. Dilating a small-caliber ureteric orifice may induce ureteral ischemia, perforation, vesicoureteric reflux, and stricture formation. Though access without presenting were observed in up to 60% of cases, active ureteral dilatation with 8–10 coaxial dilators was applied in 97% of these cases (86). In those cases with failure to access, the pediatric ureter remains narrow and inaccessible for the URS at the ureteral orifice, the iliac vessels, or the ureteropelvic junction (87).

A 9/11Fr ureteric access sheath (UAS) is applied in the majority of the procedures (5). Based on the UAS, FLURS can be removed and reintroduced repeatedly, resulting in the fragments and dust to clear and maintain a clear field of vision. Moreover, UAS allows irrigation fluid to flow, thus decreasing the intrarenal pressure. However, the use of UAS may increase the risk of ureteric injury, e.g., mucosal damage, lacerations, stricture, and even avulsion (88). As a result, it is recommended to place the post-RIRS D-J tube for a few weeks to promote the ureteric damage to heal.

In the future, with further innovation in equipment, more and more sophisticated working sheaths and flexible ureteroscopes may make it possible to treat more children's kidney stones through natural channels, which will significantly improve the safety and effectiveness of RIRS.

In a study of RIRS in treating pediatric kidney stones developed by Ekici et al. (89), the investigators accessed the ureter first by a semi-rigid ureterorenoscope and subsequently accessed the kidney by seeing through the guide with a flexible device. To avoid failure, the authors inserted a ureteral DJ stent for passive dilatation and postpone the surgery. Dilatation of the ureteral orifice is a great concern for treating pediatric urolithiasis. Active dilatation of the orifice and the corresponding procedure might cause reflux (90). Besides, it was reported that active dilatation in pediatric patients might lead to perforation (73). In addition, active dilation could also increase the number of anesthesia. Thus, the prediction of whether the ureter could be accessed in the first surgery is very important, which may both protect the pediatric patients against the complications and shorten anesthesia duration.

In some pediatric patients with multiple kidney stones and extensive distribution, a single method was commonly not enough. It was suggested that endoscopic combined intrarenal surgery (ECIRS) could achieve good surgical results (5). For example, a combined procedure could be conducted by both RIRS and PCNL methods, performed by two surgeons simultaneously. ECIRS is recommended for complete clearance of a large stone bulk, e.g., staghorn calculus and multiple stones located in difficult anatomical positions. This combined method could reduce the operating duration. Li et al. (91) performed a combined surgery by presenting with both flexible ureteroscopic lithotripsy with micro-PCNL in pediatric multiple kidney stones. The SFR in their study was 85.7% at the end of the first month after surgery, and no residual stones were seen on imaging 3 months post-operatively. The postoperative low-grade complication rate was low and no serious complications occurred. The greatest advantage of this combined technique was the reduction in the amount of surgery and anesthesia. Based on these findings, combined RIRS and PCNL may be a safe, effective, and minimally invasive operation to remove multiple renal calculi with extensive distribution in children in selected cases. Currently, there are still limited data on the outcomes of ECIRS for PU, needing further relevant studies to validate this combined method.

### Laparoscopic/robotic nephrolithotomy

For specific indications, laparoscopic can be used to treat pediatric renal and ureteric stones. In selected patients, laparoscopic and robotic-assisted surgeries (e.g., pyelolithotomy, nephrolithotomy, or ureterolithotomy) can be reasonable and safe options in children. The EAU guidelines 2021 pointed out that laparoscopic or robot-assisted surgery can be considered in children who have complicated renal anatomy (retrorenal or ectopic colon), UPJO or caliceal diverticula, megaureter, or have a history of failure of endoscopic surgery. As reported, laparoscopic pyelolithotomy in treating a ≥1 cm single stone located in an extra-renal pelvis, or the ureteric stones that were refractory to SWL or URS, can reach an SFR of 100% (92, 93).

Currently, robotic-assisted laparoscopic surgery is also one of the options in pediatric urology for its dexterity for suturing and reconstruction (94). Under the procedures, it can achieve a stone clearance up to 96% (94). It is recommended to select robotic-assisted laparoscopic surgery in treating urolithiasis with concurrent pelvi-ureteric junction obstruction, where repair and reconstruction are simultaneously being performed (95). Ghani et al. (96) performed robotic nephrolithotomy and pyelolithotomy for four children and the stones were completely removed from these patients. The future generations of robotic surgery are expected to be more inexpensive, thus it may serve as one the effective tools in pediatric urology units.

### Open nephrolithotomy

With the advent of newer minimally invasive surgery modalities, the role of open surgery for urolithiasis has been minimized. Currently, open surgery is still used in developing countries. The main reason is the low cost of treatment and high SFR. Parents from poor families, they care more about the cost and effectiveness of treatment, ignore the size of the surgical incision and the impact of the operation method on the kidneys of developing children. Rizvi et al. (97) demonstrated that there were two main reasons for selecting open surgery in a pediatric population. First, the pediatric patient's condition is one of the key factors for open nephrolithotomy. Anatomical abnormalities, complex and large stones, neglected stones with renal failure, and failed minimally invasive surgery are responsible for this open surgery. Second, the socioeconomic factors are generally specific to developing countries, e.g., the paucity of urological facilities and the residence of poor patients away from tertiary centers. Zargooshi (98) reported that 310 children underwent open surgery to treat kidney stones, and the total postoperative SFR was 95.4%. And Smaldone et al. (99) found that open surgery has become the last resort method when children suffer from severe orthopedic deformities that limit positioning for endoscopic procedures.

**Figure 1:**
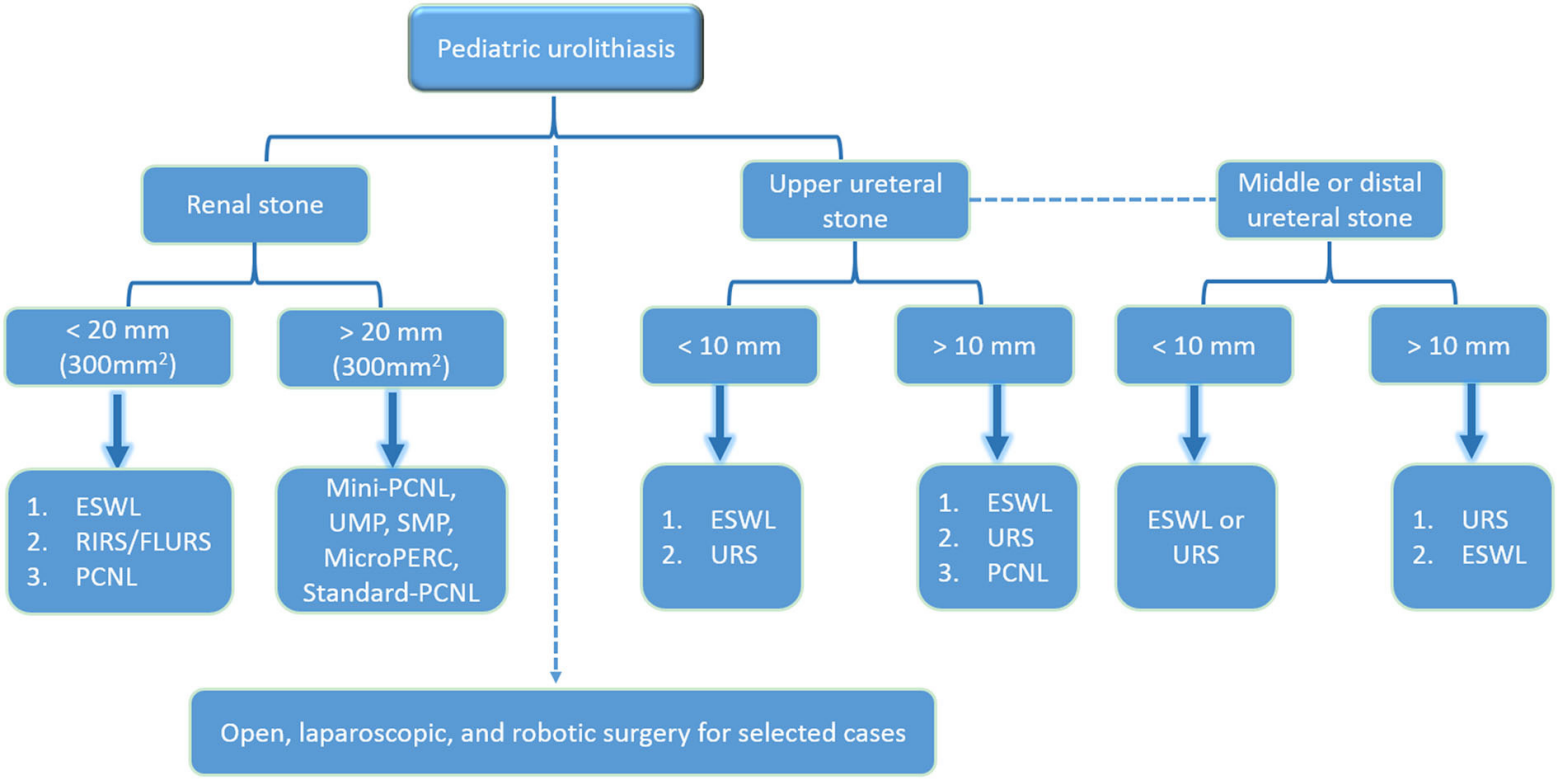
The management algorithm for renal and ureteric stones in children.

Figure 1 displays the management algorithm for renal and ureteric stones in children.

### Recent advances in pediatric stone surgery

The supine PCNL has been successfully performed in the pediatric population, which may improve the airway access and discomfort position of the children (41, 100). According to recent reports, tubeless and totally tubeless PCNL have gradually become recognized by urologists (101). These techniques might relieve pain and reduce the length of hospital stay with satisfactory surgical outcomes in children. Recently, the usage of thulium laser technology was confirmed to improve the efficacy in lithotripsy, which might be one of the promising tools for pediatric stone surgery (100).

## Conclusion

In summary, urologists should consider the peculiarity of children when treating PU. ESWL is a valuable option in the pediatric population. Modern endoscopic treatment modes together with ESWL allow individualized management of urolithiasis in the pediatric population. Future technical improvements could not only further improve the efficiency of current procedures but also concomitantly reduced the complication rates. The safety and efficacy of the PCNL procedures have been investigated in pediatric populations. It appears to be a reasonable alternative for patients with medium-to-large-sized stones, especially in those who have failed ESWL and RIRS. Further well-designed, randomized studies are still needed to better understand specific roles in the use of various miniaturized PCNL procedures. Minimizing the adverse effects on the developing kidney, reducing radiation exposure, and using a one-time treatment are the key points for treating pediatric urolithiasis. Semirigid/rigid ureteroscopes are ideal for ureteral stones, especially for lower and mid-ureter sites. Although pre-stenting may be necessary, FLURS and RIRS have also been proven to be safe and effective in children.

A comprehensive understanding of the affected factors should be taken into account by pediatric urologists to select the most beneficial treatment plan for individual children to achieve precise treatment.

## Author contributions

TP, HZ, and BH conducted the literature review and wrote the first draft of the manuscript. TP and SZ conceived the initial concept. HZ and BH revised the manuscript. All authors contributed substantially to the manuscript and approved the final manuscript.

## Funding

This work was supported by the grants from the Zhejiang Medical and Health Science and Technology Program (No. 2022RC297), the Natural Science Foundation of Zhejiang Province (No. LQ22H040009), and the Science and Technology Planning Project of Taizhou City, Zhejiang Province (No. 20ywb40).

## Conflict of interest

The authors declare that the research was conducted in the absence of any commercial or financial relationships that could be construed as a potential conflict of interest.

## Publisher's note

All claims expressed in this article are solely those of the authors and do not necessarily represent those of their affiliated organizations, or those of the publisher, the editors and the reviewers. Any product that may be evaluated in this article, or claim that may be made by its manufacturer, is not guaranteed or endorsed by the publisher.
